# Relationships Between Systemic Inflammatory Markers and 18F-FDG PET/CT Imaging and Clinical Findings in Pulmonary Sarcoidosis

**DOI:** 10.7759/cureus.36521

**Published:** 2023-03-22

**Authors:** Tugce Sahin Ozdemirel, Berna Akıncı Özyürek, Ebru Tatci, Ozlem Ertan, Esma Sevil Akkurt, Aysegul Senturk, Ozlem Ozmen

**Affiliations:** 1 Pulmonary Medicine, Ankara Ataturk Sanatorium Training and Research Hospital, Ankara, TUR; 2 Nuclear Medicine, Ankara Ataturk Sanatorium Training and Research Hospital, Ankara, TUR; 3 Pulmonology, Ankara Ataturk Sanatorium Training and Research Hospital, Ankara, TUR; 4 Pulmonology, Ataturk Sanatorium Training and Research Hospital, Ankara, TUR; 5 Pulmonology, Ankara Abdurrahman Yurtaslan Oncology Hospital, Ankara, TUR; 6 Nuclear Medicine, Ankara Ataturk Sanatorium Traning and Research Hospital, Ankara, TUR

**Keywords:** 18f-fdg, inflamation, lmr, sii index, sarcoidosis

## Abstract

Background and aim

Sarcoidosis is a multisystem inflammatory disease of unknown aetiology. This study aimed to evaluate the relationship between systemic inflammatory parameters, the systemic immune-inflammation index (SII) and the lymphocyte-to-monocyte ratio (LMR), and disease stage, clinical findings, and 18F-fluoro-2-deoxy-D-glucose (18F-FDG) tomography/computed tomography (PET/CT) uptake.

Materials and methods

Our study included 73 patients. The general characteristics, radiological features, spirometric tests, PET/CT findings, and laboratory parameters of the patients were recorded.

Results

Relapse and parenchymal fibrosis were not associated with metabolic parameters, such as LMR and SII. Serum angiotensin-converting enzyme (ACE) levels were lower in the relapsed group than in the non-relapse group. However, the patients’ PET/CT images indicated that 18F-FDG parenchym maximum standard uptake value (SUV max), lymph node SUV max, lymph node short axis dimension, SII, and LMR were similar between all patients, relapsed or not.

Conclusion

Although found to be significant in other inflammatory diseases, we found that SII and LMR alone did not indicate disease prognosis in sarcoidosis due to the small number of patients and the lack of homogeneity between the groups in our study. The usefulness of these markers for clinical use should be investigated by studies that include those with extrapulmonary sarcoidosis, and that calculate these markers at the time of disease diagnosis and during the post-treatment period.

## Introduction

Sarcoidosis is a multisystem inflammatory disease of unknown aetiology characterised by the presence of noncaseating granulomas that can involve all organs, especially the lungs and mediastinal lymph nodes. It is more common in women and young adults. The disease can be self-limiting; however, many patients experience a chronic course despite treatment. After excluding other possible pathologies, a diagnosis is made using clinical, radiological, and laboratory findings and the presence of non-caseating granulomatous inflammation [1]. When deciding on treatments, the clinical and radiological findings of patients and their spirometric values are evaluated together. However, this approach may be insufficient for predicting the severity and prognosis of inflammation in the lung [2,3].

Neutrophil, lymphocyte, and monocyte counts, which are easily obtained from a peripheral blood count, play an important role in determining inflammation. The systemic immune-inflammation index (SII) obtained from these parameters has been found to be significant in terms of disease activity and prognosis, especially in patients with COVID-19 infection, inflammatory and cardiovascular diseases, and malignancies. The SII is thought to be a good index of local immune response and systemic inflammation [4-6].

The lymphocyte-to-monocyte ratio (LMR) is a relatively new inflammatory marker that may reflect systemic inflammation. Obtained from a peripheral blood count, it is easy and cost-effective. It is used to evaluate the degree of inflammation and for prognoses in autoimmune, cardiovascular, and pulmonary diseases and malignancies [7].

In the literature, it is recommended to determine the severity of sarcoidosis and to manage the treatment by estimating the clinical course by using 18F-fluoro-2-deoxy-D-glucose (18F-FDG) positron emission tomography/computed tomography (PET/CT) [8,9].

To our knowledge, no studies in the literature have evaluated 18F-FDG PET/CT findings with systemic inflammatory markers such as LMR and SII. Our study aimed to evaluate the relationship between LMR and SII and 18F-FDG PET/CT findings as a maximum standard uptake value (SUV max), and systemic inflammatory parameters, disease stage, and clinical findings.

## Materials and methods

Patient characteristics

The medical files from our hospital records of 102 patients over the age of 18 who were diagnosed with pulmonary sarcoidosis and followed up in our clinic with endobronchial ultrasound were reviewed retrospectively. Patients who had not had pulmonary function tests (PFT) and PET/CT examinations at baseline, and who had comorbidities that could affect inflammatory markers such as autoimmune disease and malignancy were excluded from the study. The study included 73 patients who met these criteria. Participants were divided into groups with and without fibrosis, with and without relapse, male and female, and according to the stage of the disease; Inflammatory markers were evaluated in terms of PET/CT uptake values and spirometric findings. The study was approved by the Ethics Committee of the University of Health Sciences, Ataturk Sanatorium Training and Research Hospital (Date: 2022, Decision No: 2012-KAEK-15/2555).

Data collection

Data on patient demographics (age, gender), disease characteristics (duration of disease, radiographic stage, treatments), pulmonary function tests (% predicted values for forced vital capacity (FVC), forced expiratory volume at 1 second (FEV1), DLCO/VA (transfer coefficient)), blood biochemistry, and hemogram findings were retrieved from hospital records. FDG uptake by lesions in the mediastinal and lung parenchym on PET/CT images was recorded retrospectively from the hospital’s electronic information system. SII was calculated as SII = (neutrophil x platelet)/lymphocyte. The data were evaluated and recorded by the same researchers.

Statistical evaluation

The Statistical Package for the Social Sciences, Version 21.0, was used for the statistical analyses. The categorical variables were presented as numbers and frequencies. Continuous variables were assessed using the Kolmogorov-Smirnov test and histograms to determine if they had a normal or skewed distribution. Normally distributed parameters were compared using the Student’s t-test, and other variables were compared using the Mann-Whitney U test. Categorical variables were compared using chi-square or Fisher exact tests, where appropriate. The strength of the relationship between the two variables was determined using Spearman or Pearson correlation coefficients. A statistical difference was considered when p < 0.05.

## Results

A total of 73 patients diagnosed with sarcoidosis were included in the study, of which 52 (71.2%) were female, and the mean age of the patients was 48.9 ± 14.1. We observed a 22% (n = 16) relapse rate in the whole sample. Two (12.5%), 13 (81.3%), and 1 (6.2%) patients relapsed in sarcoidosis stage 1, stage 2, and stage 4, respectively (p = 0.055). Relapse occurred with a similar frequency in both genders. Relapse occurred frequently in patients treated with corticosteroids, but this failed to reach significance (p = 0.058). Relapse and parenchym fibrosis were not associated with metabolic parameters such as LMR and SII. Serum ACE levels were lower in the relapsed group than in the non-relapsed group: 38 (10-150) vs 57 (21-147); p = 0.015. As determined by the patients’ PET/CT, parenchyma SUV max, lymph node SUV max, lymph node short axis dimension, SII, and LMR were similar between relapsed and non-relapse patients. The patient characteristics of the relapse and non-relapse groups are shown in Table 1.

**Table 1:** Main characteristics of the participants LMR: lymphocyte to monocyte ratio, SII: systemic immune-inflammation index, FEV1: forced expiratory volume in 1 s, FVC: forced vital capacity, DLCO: carbon monoxide diffusion capacity, ACE: Angiotensin-converting enzyme

		Total (n=73)	No relapse (n=57)	Relapse (n=16)	p
Gender					0.128
	Female, n(%)	52 (71.2)	38 (66.7)	14 (87.5)	
	Male, n(%)	21 (28.8)	19 (33.3)	2 (12.5)	
Age, n ± SD		48.9 ± 14.1	48.3 ± 13.8	51.5 ± 15.4	0.425
Age ≥ 65 years, n(%)		11 (15.1)	9 (15.8)	2 (12.5)	0.745
Stages					0.055
	Stage 1	20 (27.4)	18 (31.6)	2 (12.5)	
	Stage 2	40 (54.8)	27 (47.3)	13 (81.3)	
	Stage 4	13 (17.8)	12 (21.1)	1 (6.2)	
Steroid use, n(%)		21 (28)	13 (22.8)	8 (50)	0.058
Parenchym suvmax, median (min-max)		6.41 (1.37-25.34)	6.86 (1.44-25.34)	5.08 (1.37-17.97)	0.748
Lymph node suvmax, median (min-max)		14.2 ± 6.8	14.3 ± 6.4	14.1 ± 8.4	0.902
lymph node short axis dimension, n ± SD		21.2 ± 6.5	21.9 ± 6.2	18.6 ± 7.3	0.078
LMR, median (min-max)		2.66 (0.73-10.19)	2.67 (0.73-6.75)	3.25 (1.29-10.19)	0.167
SII, median (min-max)		787.63 (272.31-3010.66)	759.67 (315.52-2224.40)	827.29 (272.31-3010.66)	0.709
FEV1, n ± SD		86.02 ± 19.85	85.59 ± 20.04	87.17 ± 20.13	0.818
FVC, n ± SD		93.44 ± 18.32	93.74 ± 18.56	92.66 ± 18.46	0.865
FEV1/FVC, n ± SD		80.57 ± 7.21	79.19 ± 6.26	84.56 ± 8.64	0.053
DLCO, n ± SD		93.88 ± 17.16	92.05 ± 16.13	100.80 ± 21.14	0.321
DLCO/VA, n ± SD		107.14 ± 18.35	107.18 ± 18.18	107 ± 21.95	0.176
ACE, median (min-max)		53 (10-150)	57 (21-147)	38 (10-150)	0.015

When we divided the patients into 2 groups, namely fibrosis and non-fibrosis, we found that SII and LMR did not differ significantly. The patient characteristics of the fibrosis and no-fibrosis groups are shown in Table 2. The male patients were prone to more lung fibrosis than the female patients: 7 (33.3%) vs 6 (11.5%); p = 0.042 (Table 3). Although not statistically significant, the SII value was found to be lower in stage 1 patients than in stage 2 and 4 patients (Table 4). We found no correlation between LMR, SII, parenchyma SUV max, lymph node SUV max, and measured respiratory function (Table 5).

**Table 2:** Main characteristics of the participants LMR: lymphocyte to monocyte ratio, SII: systemic immune-inflammation index

		Total (n=73)	No fibrosis (n=60)	Fibrosis (n=13)	p
Gender					0.042
	Female, n(%)	52 (71.2)	46 (76.7)	6 (46.2)	
	Male, n(%)	21 (28.8)	14 (23.3)	7 (53.8)	
Age, n ± SD		48.9 ± 14.1	48.8 ± 14.3	49.5 ± 13.7	0.878
Age ≥ 65 years, n ± SD		11 (15.1)	8 (13.3)	3 (23.1)	0.401
Steroid use, n(%)		21 (28)	16 (26.7)	5 (38.5)	0.501
Parenchym suvmax, median (min-max)		6.41 (1.37-25.34)	6.24 (1.37-25.34)	6.86 (1.89-17.58)	0.558
Lymph node suvmax, median (min-max)		14.2 ± 6.8	14.9 ± 6.6	11.2 ± 7	0.076
lymph node short axis dimension, n ± SD		21.2 ± 6.5	21.5 ± 5.9	19.3 ± 8.7	0.281
LMR, median (min-max)		2.66 (0.73-10.19)	2.7 (0.73-10.19)	2.55 (1-4.20)	0.371
SII, median (min-max)		787.63 (272.31-3010.66)	773.65 (272.31-3010.66)	809.67 (359.33-1596)	0.458

**Table 3:** Main characteristics of the participants LMR: lymphocyte to monocyte ratio, SII: systemic immune-inflammation index

		Total (n=73)	Male (n=21)	Female (n=52)	p
Age, n ± SD		48.9 ± 14.1	39.9 ± 12.4	52.6 ± 13.2	<0.001
Age ≥ 65 years, n(%)		11 (15.1)	1 (4.8)	10 (19.2)	0.160
Fibrosis, n(%)		13 (17.8)	7 (33.3)	6 (11.5)	0.042
Stages					0.053
	Stage 1	20 (27.4)	3 (14.3)	17 (32.7)	
	Stage 2	40 (54.8)	11 (52.4)	29 (55.8)	
	Stage 4	13 (17.8)	7 (33.3)	6 (11.5)	
Steroid use, n(%)		21 (28)	6 (28.6)	15 (28.8)	0.981
Parenchym suvmax, median (min-max)		6.41 (1.37-25.34)	6.52 (1.55-17.58)	6.30 (1.37-25.34)	0.951
Lymph node suvmax, median (min-max)		14.2 ± 6.8	12.8 ± 6.4	14.8 ± 6.9	0.276
lymph node short axis dimension, n ± SD		21.2 ± 6.5	22.3 ± 7.3	20.6 ± 6.1	0.315
LMR, median (min-max)		2.66 (0.73-10.19)	2.33 (0.75-6.75)	2.89 (0.73-10.19)	0.058
SII, median (min-max)		787.63 (272.31-3010.66)	787.63 (298.24-2224.40)	796.39 (272.31-3010.66)	0.908

**Table 4:** Main characteristics of the participants LMR: lymphocyte to monocyte ratio, SII: systemic immune-inflammation index

	Stage 1 (n=20)	Stage 2 (n=40)	Stage 4 (n=13)	p
Parenchym suvmax, median (min-max)	9 (9-9)	6.24 (1.37-25.34)	6.86 (1.89-17.58)	0.621
Lymph node suvmax, median (min-max)	15.17 (6.06-28.62)	12.84 (4.33-35.22)	8.69 (2.87-26.54)	0.730
lymph node short axis dimension, n ± SD	22.35 ± 5.7	21.15 ± 6.1	19.38 ± 8.7	0.077
LMR, median (min-max)	2.60 (0.73-5.80)	2.71 (1-10.19)	2.55 (1-4.20)	0.555
SII, , median (min-max)	678.84 (315.52-1456.57)	819.51 (272.31-3010.66)	809.67 (359.33-1596)	0.325

**Table 5:** Correlation analysis LMR: lymphocyte to monocyte ratio, SII: systemic immune-inflammation index, FEV1: forced expiratory volume in 1 s, FVC: forced vital capacity, DLCO: carbon monoxide diffusion capacity

		LMR	Sİİ
Lymph node suvmax	Correlation coefficients	-0.006	0.136
	p-value	0.960	0.251
Parenchym suvmax	Correlation coefficients	-0.048	0.035
	p-value	0.739	0.808
FEV1	Correlation coefficients	-0.097	0.116
	p-value	0.531	0.452
FVC	Correlation coefficients	0.060	-0.020
	p-value	0.703	0.899
FEV1/FVC	Correlation coefficients	0.049	0.036
	p-value	0.779	0.837
DLCO	Correlation coefficients	0.103	0.110
	p-value	0.631	0.609
DLCO/VA	Correlation coefficients	0.269	-0.149
	p-value	0.238	0.518

## Discussion

We investigated whether inflammatory markers, such as SII and LMR, which are reported to reflect systemic inflammation in many chronic inflammatory diseases, can determine the severity and disease activation of patients with pulmonary sarcoidosis. We found no difference between patients with pulmonary sarcoidosis divided into groups according to their stage, relapse or non-relapse, and the presence of fibrosis in terms of these markers. PET/CT imaging was not found to be associated with SUV max values. A significant negative correlation was observed between ACE and relapse. Parenchymal fibrosis was higher in men.

SII, a parameter that combines three types of inflammatory cells (lymphocytes, neutrophils, and platelets), is an improved marker for systemic inflammatory response. A high SII value has been found to be associated with disease severity and poor prognosis in many diseases and malignancies, indicating a strong inflammatory response and a weak immune response in patients [10,11]. First described by Hu et al. (2014), it was defined as a useful index for the prognosis of patients with hepatocellular carcinoma [12]. In one study, it was found that the SII in patients with ankylosing spondylitis was higher than in both healthy controls and other patients in the remission stage, and it showed a positive correlation with disease activity [13]. In another study, SII was presented as a new biomarker for demonstrating the disease activity of rheumatoid arthritis, as well as predicting rheumatoid arthritis-related interstitial lung disease [14]. However, we found no studies evaluating SII in sarcoidosis in the literature. In our study, no correlation was found between recurrence and SII, and the degree of radiological involvement. However, all the patients were stable at the time of diagnosis; therefore, we suggest that it would be more significant if the SII value could be found in the medical records and compared in those who had recurrence during the follow-up and started treatment and when this treatment was started.

The literature indicates that LMR is an independent factor that affects disease activity in rheumatoid arthritis [7]. A study showed a relationship between LMR and lung function and blood gas analysis in patients with pneumoconiosis and indicated that it can be a sensitive indicator in the evaluation of pneumoconiosis [15].

High ACE levels have been reported in 56%-61% of sarcoidosis patients. Used in disease activity assessment and treatment planning, it has been shown to be unrelated to disease severity, progression, and treatment response [16-18]. In another study, significant correlations were found between high ACE levels and relapse and clinical course [19]. In our study, it was found to be higher in non-relapse cases.

Chest radiography is the first imaging method to be used in the evaluation of pulmonary sarcoidosis, but radiological scoring is insufficient to determine inflammatory activity. While CT allows for a detailed anatomical examination, it cannot show the activity of inflammatory disease. Therefore, in recent years, the usefulness of PET/CT in the staging of sarcoidosis, activity determination, and the evaluation of response to treatment has been studied. Inflammatory cells, especially neutrophils and macrophages, are activated by cytokines released by inflammation. In these activated cells, the expression of glucose transporters increases, and there is an increase in the entry of 18F-FDG into the cells [9,20]. When determining new inflammatory exacerbations developing in chronic sarcoidosis, serological examinations are negative; therefore, FDG PET/CT can be used to evaluate inflammation in symptomatic patients and in stage 4 sarcoidosis patients with parenchymal fibrosis. PET/CT is mainly used in the diagnosis and follow-up of malignant lesions; an SUVmax value of less than 2.5 suggests a benign lesion. Many studies have shown increased FDG uptake in sarcoidosis. [21-23]. However, 18F-FDG uptake in sarcoidosis is nonspecific and is a false-positive condition. In our study, mediastinal lymphadenopathy SUVmax (14.2 ± 6.8) and lesion SUVmax in the lung (6.41 (1.37-25.34) were found to be high. PET/CT can be a guide when determining the extent of intrathoracic disease, detecting the presence of active disease, detecting extrathoracic sarcoidosis involvement areas, and evaluating responses to treatment in patients with a sarcoidosis diagnosis. However, in our study, it was not found to be associated with disease relapse, disease stage, or inflammatory markers.

The weakness of our study may be that it was retrospective, the data was obtained from file records and it only included patients with pulmonary sarcoidosis.

## Conclusions

Although SII has been found to be significant in other inflammatory diseases, we found that SII and LMR alone did not indicate disease prognosis in sarcoidosis. This was due to the small number of patients and the lack of homogeneity between the groups in our study. Therefore, their use as stand-alone markers does not seem appropriate. Future studies should investigate the usefulness of these markers for clinical use by including patients with extrapulmonary sarcoidosis. This should be carried out by calculating these markers at the time of disease diagnosis and in the post-treatment period.

## References

[1] Llanos O, Hamzeh N (2019). Sarcoidosis. Med Clin North Am.

[2] Spagnolo P, Rossi G, Trisolini R, Sverzellati N, Baughman RP, Wells AU (2018). Pulmonary sarcoidosis. Lancet Respir Med.

[3] Papiris SA, Manali ED, Pianou NK (2020). 18F-FDG PET/CT in pulmonary sarcoidosis: quantifying inflammation by the TLG index. Expert Rev Respir Med.

[4] Huang H, Liu Q, Zhu L, Zhang Y, Lu X, Wu Y, Liu L (2019). Prognostic value of preoperative systemic immune-inflammation index in patients with cervical cancer. Sci Rep.

[5] Fois AG, Paliogiannis P, Scano V (2020). The systemic inflammation index on admission predicts in-hospital mortality in COVID-19 patients. Molecules.

[6] Wei L, Xie H, Yan P (2020). Prognostic value of the systemic inflammation response index in human malignancy: a meta-analysis. Medicine (Baltimore).

[7] Li M, Xie L (2021). Correlation between NLR, PLR, and LMR and disease activity, efficacy assessment in rheumatoid arthritis. Evid Based Complement Alternat Med.

[8] Sobic-Saranovic D, Artiko V, Obradovic V (2013). FDG PET imaging in sarcoidosis. Semin Nucl Med.

[9] Jamar F, Buscombe J, Chiti A (2013). EANM/SNMMI guideline for 18F-FDG use in inflammation and infection. J Nucl Med.

[10] Yang R, Chang Q, Meng X, Gao N, Wang W (2018). Prognostic value of systemic immune-inflammation index in cancer: a meta-analysis. J Cancer.

[11] Gürol G, Çiftci İH, Terizi HA, Atasoy AR, Ozbek A, Köroğlu M (2015). Are there standardized cutoff values for neutrophil-lymphocyte ratios in bacteremia or sepsis?. J Microbiol Biotechnol.

[12] Hu B, Yang XR, Xu Y (2014). Systemic immune‐inflammation index predicts prognosis of patients after curative resection for hepatocellular carcinoma. Clin Cancer Res.

[13] Wu J, Yan L, Chai K (2021). Systemic immune-inflammation index is associated with disease activity in patients with ankylosing spondylitis. J Clin Lab Anal.

[14] Xu Y, He H, Zang Y (2022). Systemic inflammation response index (SIRI) as a novel biomarker in patients with rheumatoid arthritis: a multi-center retrospective study. Clin Rheumatol.

[15] Hu XX, Liu SP, Zhou RS, Hu MN, Wen J, Shen T (2022). [Correlation analysis between blood routine-derived inflammatory markers and respiratory function in pneumoconiosis patients]. Article in Chinese. Zhonghua Lao Dong Wei Sheng Zhi Ye Bing Za Zhi.

[16] Kıter G, Müsellim B, Çetinkaya E (2011). Clinical presentations and diagnostic workup in sarcoidosis: a series of Turkish cases. Tüberküloz ve Toraks Dergisi.

[17] (1999). Statement on sarcoidosis. Joint Statement of the American Thoracic Society (ATS), the European Respiratory Society (ERS) and the World Association of Sarcoidosis and Other Granulomatous Disorders (WASOG) adopted by the ATS Board of Directors and by the ERS Executive Committee, February 1999. Am J Respir Crit Care Med.

[18] McGrath DS, Foley PJ, Petrek M (2001). Ace gene I/D polymorphism and sarcoidosis pulmonary disease severity. Am J Respir Crit Care Med.

[19] Belhomme N, Jouneau S, Bouzillé G (2018). Role of serum immunoglobulins for predicting sarcoidosis outcome: A cohort study. PLoS One.

[20] Ahmed N, Kansara M, Berridge MV (1997). Acute regulation of glucose transport in a monocyte-macrophage cell line: Glut-3 affinity for glucose is enhanced during the respiratory burst. Biochem J.

[21] Cook GJ, Fogelman I, Maisey MN (1996). Normal physiological and benign pathological variants of 18-fluoro-2-deoxyglucose positron-emission tomography scanning: potential for error in interpretation. Semin Nucl Med.

[22] Brudin LH, Valind SO, Rhodes CG, Pantin CF, Sweatman M, Jones T, Hughes JM (1994). Fluorine-18 deoxyglucose uptake in sarcoidosis measured with positron emission tomography. Eur J Nucl Med.

[23] Gotway MB, Storto ML, Golden JA, Reddy GP, Webb WR (2000). Incidental detection of thoracic sarcoidosis on whole-body 18fluorine-2- fluoro-2-deoxy-D-glucose positron emission tomography. J Thorac Imaging.

